# Bio Discarded from Waste to Resource

**DOI:** 10.3390/foods10112652

**Published:** 2021-11-01

**Authors:** Irene Dini

**Affiliations:** Department of Pharmacy, University of Naples Federico II, Via Domenico Montesano 49, 80131 Napoli, Italy; irdini@unina.it

**Keywords:** food waste, recycling, nutrient, bioactive molecules, analytical procedures

## Abstract

The modern linear agricultural production system allows the production of large quantities of food for an ever-growing population. However, it leads to large quantities of agricultural waste either being disposed of or treated for the purpose of reintroduction into the production chain with a new use. Various approaches in food waste management were explored to achieve social benefits and applications. The extraction of natural bioactive molecules (such as fibers and antioxidants) through innovative technologies represents a means of obtaining value-added products and an excellent measure to reduce the environmental impact. Cosmetic, pharmaceutical, and nutraceutical industries can use natural bioactive molecules as supplements and the food industry as feed and food additives. The bioactivities of phytochemicals contained in biowaste, their potential economic impact, and analytical procedures that allow their recovery are summarized in this study. Our results showed that although the recovery of bioactive molecules represents a sustainable means of achieving both waste reduction and resource utilization, further research is needed to optimize the valuable process for industrial-scale recovery.

## 1. Introduction

Bio-waste residues include food waste and agricultural, forestry, marine, and animal-derived residues. Within the context of a more circular economy, these wastes are recategorized as raw materials, feedstock, or energy. These wastes are not explicitly covered within European legislation, except for food waste, regarding targets for separation and reduction. However, Europe provides Research and Development funding for the EU bio-economy sector. Horizon 2020 resources assigned to this initiative amounted to €3.7 billion in 2014–2020, of which €90 million was used for proposals related to bio-waste [1]. The Member States are urged to reduce biodegradable waste entering landfills, as required by the Landfill Directive. Food industries, hospitality, and households produce food waste (FW). Food is biological material that is subject to degradation.

Half of all food grown (close to 1.3 billion t) [2] is lost or wasted before and after reaching the customer [3]. Food loss occurs when production exceeds demand, farmers harvest crops prematurely, and as a result of inadequate sales and storage conditions (e.g., imperfections in packaging), safety issues, and contamination [4]. FW is expected to rise over the next 25 years due to economic and demographic growth, mainly in Asian countries. The annual volume of FW in Asian countries may increase from 278 to 416 million t from 2005 to 2025 [5]. In recent decades, the magnitude of global “waste” has been correlated to malnutrition and pollution. The carbon footprint of food waste contributes to greenhouse gases (it causes the release of 3.3 billion t of CO_2_ into the atmosphere annually) [6] and dioxins [7], which may lead to several environmental problems. Therefore, it is a moral and economic challenge to recycle waste to meet human or livestock needs and minimize the environmental problems related to its disposal. A significant reason for the low recycling rate is the low disposal cost compared to the recycling/conversion cost. The European Parliament and Council started the Circular Economy Package in 2018 to obtain efficient food supply chains [1]. The objective is a world without waste, with a responsible attitude towards products, materials, resources, and the environment. Such actions require that food waste management strategies are urgently considered, and social and behavioral solutions for enhancement are discussed. Several technological solutions have been proposed, such as developing collection systems for mixed biodegradable waste anaerobic digestion, composting, and incineration [1]. An Expert Working Group has been created on Food Losses and Food Waste to make policy initiatives and enhance EU legislation, programs, and policies on food waste prevention with the aim of halving food waste by 2050.

To achieve this goal, the Member States must establish food waste prevention measures and uniform measurement methodologies. An effective means of managing food waste is to produce biochar or bioenergy (e.g., biogas, biodiesel), or to extract primary and secondary metabolites to use in cosmetics, pharmaceuticals, and food supplements [8,9,10,11,12,13]. The technological developments in the chemical, physical, and biological treatments of food waste and their potential applications within a sustainable bioeconomy are summarized in this work. The articles published in recent years in the peer-reviewed journals in Scopus, Web of Science, and Google scholar were investigated to achieve this goal. The areas of focus of the published reviews and scientific articles are identified and cited accordingly. The results of the published data are compared, and suggestions are given.

## 2. Biochar

Biochar (char, charcoal, or agrichar) is a stable nonfossil-based carbonaceous product made from the thermochemical (torrefaction (dry or wet), pyrolysis, gasification, or hydrothermal processing) conversion of biomass [14], which helps to improve soil fertility in an environmentally friendly way through the development of biocomposite [15,16,17], as well as being used in green concrete production [18]. Biochar has variable performance in terms of the functioning of its biosource and the process used to make it. Pyrolysis is a facile and low-cost process that allows solid (biochar), liquid (bio-oil), and gas (syngas, e.g., hydrogen carbon dioxide and nitric oxide) products to be made [19]. It is performed at variable temperature (300 to 900 °C) for several seconds (fast pyrolysis) or hours (slow pyrolysis) without oxygen. Slow pyrolysis produces more yields of biochar than rapid pyrolysis [20]. The gasification produces solid, liquid, and mainly gas products, partially oxidizing the feedstock with oxygen, air, steam, etc., at a temperature higher than 700 °C. The pyrolysis and gasification usually proceed without water. The hydrothermal carbonization is performed in a reactor at a temperature below 250 °C [21]. The flash carbonization converts the feedstock into solid and gas products in around 30 min with a controlled pressure (1–2 Mpa) and variable temperature (300 to 600 °C) [22]. The torrefaction converts feedstock into hydrophobic solid products, removing oxygen and moisture at 200 to 300 °C [23]. Temperature, retention time, heating rate, and air conditions affect biochar’s physiochemical properties [24]. Chemical (acidification, alkalinization, oxidation, and carbonaceous materials modification) and physical modifications (gas and steam purging) can improve biochar’s environmental performance [25]. The surface area is improved by alkaline, stem, gas, and carbon material modifications. The ratio of carbon, nitrogen, and oxygen affects biochar’s properties. The basic nature of biochar is subject to the ratio of nitrogen to carbon. The hydrophilic properties depend on the ratio of oxygen to carbon [25]. Biochar has been employed to remediate organic pollutants by means of hydrogen binding, surface complexation, electrostatic attractions, and pi–pi and acid–base interactions [26], and the heavy metals in soil by precipitation and surface complexation chemical reduction, cation exchange, and electrostatic attraction [26]. Moreover, biochar can improve cation exchange capacity, neutralize acidic soil, and enhance soil fertility [27,28]. Recent studies have shown biochar’s great potential to improve the decomposition of organic solid waste by offering habitats and favorable growing conditions for microorganisms [29] and removing pollutants (i.e., antibacterial drug) from water and wastewater [30,31].

## 3. Bioenergy (Biogas, Bioalcohol, Biodiesel, and Bioelectricity)

The global market value of bioenergy is approximately US $25.32 billion and is expected to increase by US $40 billion by 2023. Waste is transformed into bioenergy by biological (e.g., anaerobic digestion, fermentation, esterification, and electro fuel cells) and physicochemical methods (e.g., pyrolysis, incineration, gasification, and landfills) [32,33,34,35].

Microbial communities produce biogas by anaerobic digestion [36,37]. Reactions of the triacylglycerols’ esterification/transesterification with alcohols and enzymes or chemical catalysts allow biodiesel’s production [38,39,40,41]. Microbial fuel cells and fermentation provide bioelectricity and bioalcohol [42]. The productivity of the biological process used to convert biowaste into energy is affected by regional climatic conditions and the elevated cost of the solvent used to extract triacylglycerol for the production of biodiesel and alcohol in order to make bioalcohol [43,44].

Various strategies were used to pretreat the biowaste, according to their origin (e.g., agro-industry, municipal waste, and animal waste), before conversion into bioenergy. Biowastes composed of hemicellulose, cellulose, and lignin need physical, chemical, physicochemical, or biological pretreatment to make carbohydrate polymers available to hydrolases [41]. Animal waste must be ground uniformly and exposed to high temperatures (115–145 °C) to release fat [45]. Cooking oil must be filtrated, distillated (to eliminate water), and adsorbed to remove free fatty acids produced during the frying process [46]. Waste enriched with salt and heavy metals must be subjected to electrodialysis [47] or activated carbon adsorption [48]. Regarding technological solutions used to convert biowaste into bioenergy, various biological methods (e.g., transesterification, anaerobic digestion, microbial fuel cells, and fermentation) and physicochemical methods (e.g., incineration, landfill, gasification, and pyrolysis) have been used [38,49,50]. Biogas is produced via the anaerobic (without oxygen) digestion of microorganisms under controlled pH and temperature conditions. Four steps are performed to obtain gas: hydrolysis (hydrolases convert biomass into amino acids, sugars, and fatty acids), acidogenesis (acidogenic bacteria convert these molecules into fatty acids, CO_2_, and H_2_), acetogenesis (acetogenic bacteria convert the latter into acetic acid), and methanation (methanogenic bacteria convert all the intermediate products into methane, water, and CO_2_) [51,52]. The biodiesel is produced by transesterifying animal fat, vegetable oil, or microbial oil (using basic, acidic, and enzymatic catalysts) in alcohols [32,53] before extracting them with chemical, mechanical, supercritical fluid, enzymatic, microwave-assisted, or accelerated solvent extraction processes [54,55]. Alcohol is produced via the fermentation of biowaste, which is mainly obtained from food crops for security reasons [56]. Bioelectricity is produced through the use of microbial fuel cells under anaerobic conditions [57,58]. *Saccharomyces*, *Aeromonas*, Escherichia, Candida, Clostridium, Shewanella, and *Klebsiella* are microbes that are able to produce electricity in a microbial fuel cell [59,60,61,62]. An exogenous mediator can enhance a microbial fuel cell’s performance and decrease microbial growth, but it is toxic. 

## 4. Recovery of Bioactive from Food Waste

Biowastes, especially food wastes, contain bioactive compounds that are suitable for producing functional foods, supplements, and nutricosmetics [63,64,65,66,67]. Vegetables and fruits have primary metabolites (e.g., amino acids, lipids, dietary fibers, cellulose, hemicellulose, lignin, and fatty acids) [68,69,70], and secondary metabolites (e.g., flavonoids, phenols, alkaloids, glucosinolates, carotenoids, and terpenes) [71]. The extraction of bioactive compounds from biowastes depends on the source, functionality, chemical properties, and end-use. Various temperatures, pH values, electromagnetic waves, and extraction techniques are used (e.g., supercritical fluid, subcritical water, ultrasonic wave, microwave, and pulsed electric field) [72]. One of the oldest approaches used to obtain bioactive molecules from biowaste at research and industrial levels is solid-state fermentation [73]. Solid-state fermentation (SSF) uses micro-organisms grown on solid substrates without an open liquid [74]. It employs fungi or bacteria (specific strains or mixed culture) to obtain the maximum nutrient attention from the substrate for fermentation. In the SSF, the substrates (e.g., byproducts of cassava, grains, potato, sugar beet pulp, beans, etc.) used as a nutrient source [75] are solid or soaked (sugars, lipids, organic acids, etc.) with a liquid medium [76]. The SSF contributes to high volumetric productivity by increasing product concentrations and reducing effluent production (e.g., N_2_O, CH_4_, and NH_3_) [76]. It improves the functional properties of the solid substrates that originated from agro-industrial wastes that affect proteins’ physicochemical properties (e.g., solubility) and structures [77,78]. The fungi used in SSF transform proteins with many amino acids into proteins with few units, improving the substrate’s solubility in the water system [78]. Solid-state fermentation improves the water and oil binding properties affecting the hydrophobic and hydrophilic domains of the solid substrates’ components [79,80] and entrapping water and oil against gravity after opening the protein structures. Moreover, SSF enhances the cohesive nature of the proteins by forming large air cells [81] and influences the emulsion stabilizing and forming properties that alter the solid substrate’s solubility, molecular flexibility, and surface hydrophobicity [82]. SSF was used to extract protein from pumpkin, potato, cabbage, cauliflower, and brinjal [83], protease from vegetable waste [84], lycopene from tomato waste [85], and phenolics from rice bran [86]. Liquid fermentation (or submerged fermentation (SmF)) is mainly used in industrial processes since it has low cost, high yield, and little contamination. Water or energy requirements and physical space are some disadvantages of this technology [86]. SmF was used to obtain the enzyme pectinase from fungi [87] and agro-wastes [88,89,90,91], and exo-polygalacturonase from orange peel [92]. 

### 4.1. Innovative Processes Used to Extract Bioactive from Food Waste

#### 4.1.1. Supercritical Fluids Extraction

Supercritical fluids have a higher solute capacity, diffusivity, and lower viscosity than other solvents since they, similarly to gases, quickly diffuse into a solid matrix and, in a comparable manner to liquids, dissolve compounds. Therefore, extraction with supercritical fluids produces better yields in shorter extraction times than extraction with other solvents [93]. In the separators, the solid (e.g., bioactive, etc.) is stored at the bottom, and fluid is discharged into the environment or recycled [94]. Supercritical CO_2_ is mainly employed to extract nonpolar or partially polar bioactive molecules from food byproducts under temperature, and pressure-controlled conditions (usually T = 31 °C and P = 74 bar) as CO_2_ is non-toxic, non-explosive, and it is easily removed from the finished product [95,96]. CO_2_ is used with a co-solvent or a modifier to improve the solvation power of biomolecules in the solid matrix [97]. This method employs large volumes of organic solvents. Therefore, supercritical antisolvent extraction methodology was proposed to reduce the consumption of organic solvents. The solvent is completely miscible in the supercritical antisolvent, the solute precipitates as a powder, and the liquid is extracted. Unfortunately, molecules that are soluble or partly soluble in CO_2_ are discharged [98]. The influence of temperature and pressure on extraction performance varies according to the material type, origin, and target compound. The mixture’s critical point indicates the temperature, pressure, and composition at which the mix (CO_2_–organic solvent) is supercritical. Supercritical antisolvent extraction methodology has been used to fractionate amino acids extracted with ethanol from tobacco leaves [99] and phospholipids from soybean oil [100]. Slow extraction kinetics limit the use of supercritical antisolvent extraction methodologies [101]. The combined use of ultrasound or enzyme enhances the extraction efficiency [72].

#### 4.1.2. Supercritical Water Extraction

Subcritical water extraction involves the heating of water (T= 100–320 °C) at a controlled pressure (~20–150 bar) to enhance the dissolution of nonpolar molecules. At these conditions, the dielectric constant of water decreases (~27 at 250 °C), becoming comparable to that of methanol and ethanol (33 and 24, respectively, at 25 °C), together with the viscosity, polarity, and surface tension and improves the nonpolar molecules dissolution [102]. This technology was employed to extract phenolics from onion [103] and kiwi [104], and lipids [105] and phenolics [106] from red wine grape pomace. Pretreatments with ultra-sonication, microwaves [107], and gas hydrolysis (N_2_ or CO_2_) accelerate the extraction time [72]. The water’s high reactivity and corrosiveness (at a subcritical state) limit this technology’s use [108]. 

#### 4.1.3. Pressurized Liquid Extraction

Pressurized liquid extraction uses elevated temperature and pressure to improve the performance of traditional liquid extraction techniques [109]. The high temperatures disrupt the analyte–sample matrix interactions (due to hydrogen bonding, van der Waals forces, and dipole attraction) [110], and improve the solvent wetting of the sample (reducing the surface tension of the solutes, matrix, and solvent) [111] and the diffusion of the molecules into the solvent. High temperatures’ disadvantages include poor extraction selectivity, disintegration, and hydrolytic degradation of the thermo-labile compounds [112,113]. The high pressures facilitate the analyte extraction, thereby facilitating contact between the solvent and the analytes, controlling the air bubbles within the matrix, disrupting the matrix, and forcing the solvent into the matrix pore [114]. Water is used to pressurize hot water extraction (PHWE) or extract subcritical water (SWE). SWE was previously used to extract phenolics from biowaste [115]. 

#### 4.1.4. Ultrasound-Assisted Extraction

Ultrasound-assisted extraction employs the frequencies of the ultrasonic region (20 kHz to 100 kHz) to extract biomolecules from biomaterials. Humans cannot detect the frequencies that determine vibration, acoustic cavitation, and mixing effects in liquid media. The physical forces of the ultrasonic waves determine shockwaves, microjets, and turbulence, which destroy cell walls, facilitating the extraction of biomolecules [116,117]. Acoustic cavitation enhances the coalescence of multiple bubbles and mass accumulation in the bubble. The bubbles initially grow and successively collapse when they reach a critical size (resonance). The resonance is inversely related to the applied frequency and directly related to temperature [118]. The cavitation intensifies the movement of the solvent (e.g., water, methanol, ethanol, and hexane) into the cell-matrix and the extraction of the biomolecules. The ultrasound-assisted extraction uses shorter times, enhances the extraction rate, and provides a higher yield than conventional techniques [72]. Longer extraction times can cause undesirable changes in the extract [72]. Phenolics from the pomegranate peel [119] and grape pomace [120] were extracted by ultrasound-assisted extraction. 

#### 4.1.5. Microwave-Assisted Extraction 

Microwave-assisted extraction is used in combination with solvent extraction to improve yields, and to reduce the solvent volumes and extraction times [121,122,123]. The polar materials absorb the microwave energy and turn it into heat by dipole rotation and ionic conduction. The ranges of the electromagnetic field vary from 300 MHz to 300 GHz. Solvents with a high dielectric constant are used to improve the extraction efficiency of this technique [124]. Each microwave system consists of a source, waveguide (magnetron), and applicator. The magnetron contains a vacuum tube with an electron-emitting cathode and anode coupled by the fringing fields. The magnetic field strength and tube current control the magnetron’s power output. The transmission lines and waveguides regulate the electromagnetic wave. The waveguide can be used for microwave heating when wall slots introduce the material, and a matched load terminates the waveguide (traveling wave devices).

Alternatively, the microwaves can be irradiated by slot arrays or horn antennas of waveguides (standing wave devices) [125]. The solvent type, irradiation time, microwave power, and extraction temperature affect the performance of MW extraction [126]. The solvent must be able to solubilize the bioactive molecules (with a Hildebrand solubility parameter similar to those of the extracting compounds) [127] and absorb microwave energy (polar solvents with a high dielectric constant such as water and ethanol have a better capacity to absorb electromagnetic energy and sell it as heat) [128]. The industrial scale-up process is realized in apparatuses that are capable of withstanding high pressures, which constitute appropriate reaction vessels [129]. Alkaloids from Macleaya cordata [130], polyphenols from rice [131] and roselle [132], oligosaccharides from food waste [133], and pectins from citrus [134] were extracted using this technology.

#### 4.1.6. Microwave-Assisted Enzymatic Extraction

Microwave-assisted enzymatic extraction involves microwaves and a mixture of enzymes (e.g., pectinase, celucast, and viscozyme) to disintegrate fruit and vegetable matrices and improve the extraction of biomolecules. The synergetic effect of microwaves and more than one enzyme decreases the enzyme cost and improves yields [135]. This technology was used to extract fish protein hydrolysates from rainbow trout [136], phenolics and anthocyanins from soybean [137], and oligosaccharides from American Cranberry [138].

#### 4.1.7. Pulsed Electric Field Extraction

Pulsed electric field extraction is a nonthermal method that applies moderate electric field strength for some milli- or nanoseconds to destroy wall cells [139]. The matrix exposed to electric fields collects charges on any side of the membrane surface, making transmembrane potential and pores when the transmembrane potential reaches the critical limit into the weaker sections of the membrane (cell electroporation) [140]. The cell electroporation improves the intracellular compounds’ release and, consequently, their extraction yields [141,142]. Elective field intensity, pulse number, specific energy input, and treatment temperature affect the pulsed electric field extraction. Electric field intensity influences the cell membranes’ electroporation [143]. Pulsed electric field extraction is completed when the strength and electric field’s applied voltage are above the critical transmembrane potential. The electroporator is an electrical system used for the extraction process. It comprises a pulsed power modulator (which offers the high-voltage pulses to the treatment chamber) [144], a treatment chamber, and a control unit [145]. Power switches transfer the stored energy reasonably economically [146]. Problems related to using this technique are solvent electrolysis and electrode corrosion, which can occur when the electric field strength is high enough. The electrochemical reactions can produce metallic ions and reactive oxygen species. Metallic ions can catalyze the decomposition of hydrogen peroxide into hydroxyl radicals via the Fenton reaction [147,148]. It is possible to produce a pretreatment or use a continuous-flow treatment chamber to achieve a solid–liquid extraction. This technology, which is helpful in the extraction of heat-sensitive compounds [149], is used to extract bioactive molecules from eggshells [150], tomato juice [151], fishbone [152] wastes produced in the cooking oil industry [153], and pectin from the sugar-beet [154]. The main limitations of this technology are its deficiency of reliable and more practical electrical systems, the fouling and corrosion of the electrodes in the treatment chamber [145], the lack of knowledge of different foods’ specific internal energy (J/kg) efficiencies, and its high electricity consumption levels (which promotes CO_2_ emissions) [155]. 

#### 4.1.8. High Voltage Electrical Discharges 

The high voltage electrical discharges (HVED) technique is a nonthermal technology based on the electrical breakdown in liquids, which produces shock waves and free radicals. During the treatment, a gas pumped into the reactor ionizing forms cold plasma, and an electric field enhances the cell membrane’s permeability and improves the release of metabolites [156]. The high intensity (~10 kA) and voltage (30–40 kV) obtained by short pulses (μs–ms) between two electrodes are helpful to maximize the extraction [149]. This technology was employed to extract bioactive compounds from pomegranate peel [157], peanut shells [158], sesame cake [159], orange peel [160], and grapefruit peels [161]. 

#### 4.1.9. Liquid Biphasic Flotation

The solvent sublation and aqueous two-phase extraction system are involved in liquid biphasic flotation [162]. The biphasic flotation equipment comprises a glass column, a sintered disk, and a compressed air system [163]. Bubbles produced with air that is compressed into the glass column are used to adsorb the active compounds. The surface hydrophilicity and hydrophobicity of biomolecules affect the adsorption level [164]. The top and bottom layers of the column contain lower and higher polarity molecules, respectively [163].

### 4.2. Primary Metabolites Recovered from Agri-Food 

#### 4.2.1. Proteins

Proteins are macronutrients with a central role in human nutrition. They are formed by amino acid units. Proteins from food waste sources are used as value-added ingredients and/or products, including human foods and animal feed. The high-value products are used as foaming, thickeners, and gel stabilizers [165], and the low-value products are used as fish and animal feed [166]. Food waste protein sources can be classified into animal and plant sources. Plant byproducts used as protein sources include oat, rice, wheat bran protein [167,168,169], and defatted meals from the oil industry. Wheat bran contains between 13% to 18% of proteins [167], the defatted meals obtained from the oil industry (e.g., canola, sunflower, palm, rapeseed, and peanuts) have between 15% to 50%, and soybean curd residue contain 27% protein [170]. Sugar beet and mushroom flakes are used as a feed ingredient source since they contain 40% essential amino acids [171]. Finally, food waste proteins obtained by animals (e.g., meat, fishmeal, bone meal, yogurt, and cheese) are considered good-quality protein sources that are of high biological value [172]. Some extraction methods were used to isolate protein, including enzyme-assisted, cavitation-assisted, ultrasound-assisted, hydrodynamic cavitation, microwave-assisted, supercritical, liquid biphasic flotation, and hybrid extractions [173]. In enzyme-assisted extraction, the protein recovery depends on the enzyme ratio, substrate characteristics, extraction time, and pH [174]. Protein isolates were generally obtained by defatted pressed legume cakes and animal sources via precipitation at the isoelectric point [175]. Hydrolysate from protein isolates is also used [176,177,178] since it produces higher solubility products and smaller peptides [178,179]. Cavitation-assisted extraction is used in large-scale protein extraction. Low frequency (20 to 100 kHz), temperature, sonication power, and treatment time affect the protein yield [180]. Ultrasound-assisted extraction is coupled with enzyme-assisted or microwave-assisted extraction technologies to improve protein extraction efficiency [176]. Microwave-assisted extraction of proteins can depend on nonuniform temperature distribution and closed- or open-type vessel systems [181,182]. It enhances the proteins’ functional properties (e.g., water absorption, emulsifying, foam activity, and foam stability indexes) [176]. Supercritical extraction of proteins depends on temperature [183] and solvent concentration [184]. Chemical dehydration and/or evaporation are required to remove moisture. These procedures can affect protein purity [176]. Liquid biphasic flotation has high separation efficiency and determines the minimal protein loss [163,185]. Cell receptors, drug residues in food, and wastewater treatments were extracted using this technology [186]. 

##### Possible Uses of the Recovered Proteins

The food waste proteins can be utilized in feed supplements to enhance the food products’ functional properties [187]. Milk protein and whey protein are used to enrich ice cream [188], improve the mixture’s viscosity, and decelerate the melting time [189]. The animal proteins can be used as a foaming agent with recycled PET aggregates to produce cementitious concrete composites [190]. Whey protein can be employed to produce plastic films for food packaging materials [191].

#### 4.2.2. Pectins

Pectins are polysaccharides that are formed by d-galacturonic acid, d-galactose, or l-arabinose units, and are found in the cell walls of plant tissue [192]. The degree of pectin esterification affects the pectins’ functional properties as a thickening and gelling agent. Conventional (e.g., extraction with the mineral acids) and innovative techniques (e.g., ultrasound- or enzyme-assisted microwave- extraction) were used to extract them from biowaste. Traditionally, pectin is extracted via continuous stirring with water that is acidified (e.g., in nitric, 0.05–2M sulfuric, phosphoric, hydrochloric, or acetic acid) for 1 h under controlled temperature (80 and 100 °C) [193]. The maximum pectin yield is obtained using hydrochloric acid at pH 2.0 [194]. Innovative extraction methods help in the extraction of pectins, disrupting the cell membrane’s structure by electromagnetic or sound waves and facilitating the contact between solvent and bioactive molecules. Among the most innovative approaches, ultrasound-assisted technology improves (+20%) the pectins’ molecular weight and extraction yield compared to the traditional method under the same temperature, pH, and time conditions [195]. The microwave-assisted extraction of pectins is affected by the weight of the biomaterial, the power of the wave, the time of extraction, and the pH. For example, the optimum processing conditions to extract pectins from lime bagasse are a sample weight of 6 g, a wave power of 400 W, a time of extraction of 500 s, and a pH of 1 [196]. Finally, enzymes can enhance the extraction process by hydrolyzing the plant cell wall matrix (enzyme-assisted extraction). The enzymes used to extract pectins are protease, cellulase, alcalase, hemicellulase, xylase, α-amylase, polygalacturonase, b-glucosidase, endopolygalacturonase, neutrase, and pectinesterase [197]. 

##### Possible Uses of the Recovered Pectins

The food industry employs pectins as emulsifiers, stabilizers, thickeners, and gelling agents. 

The pharmaceutical industry uses them as drug-controlled release matrices and prebiotic, hypoglycemic, hypocholesterolemic, and metal-binding agents [198]. 

Finally, the functionalization of pectins with nanomaterials and phenolics can produce active packaging films with antimicrobial properties [199]. 

#### 4.2.3. Omega-3 from Fish Waste

Omega-3 fatty acids (e.g., eicosapentaenoic acid (EPA) and docosahexaenoic acid (DHA)) have the first double bond on carbon 3, counting from the terminal carbon. Fish are a good source of omega-3. They accumulate them from plankton, algae, and prey fish [200]. The omega-3 fatty acids regulate cell membranes’ architecture and permeability, produce energy and eicosanoids, and modulate the human body’s pulmonary, cardiovascular, immune, reproductive, and endocrine systems [200]. Their potential health benefits include the prevention of cancer, cardiovascular disease (CVD), Alzheimer’s disease, depression, rheumatoid arthritis, attention deficit hyperactivity disorder (ADHD), dry eyes, and macular degeneration [201]. Numerous apparatuses and techniques were proposed to extract omega-3 fatty acids from fishes. Traditional extraction techniques use organic solvents (e.g., hexane, methanol, petroleum ether, and chloroform), which cannot be employed on an industry scale [202,203]. Soxhlet extractor, ultrasounds, or microwave-assisted extractions decrease the time and use of solvents [204]. On an industrial scale, fish oil extraction is achieved through a wet-reduction or wet-rendering process [205]. Supercritical fluid extraction (SFC) [206] solves the problem of n-hexane use for extraction in traditional extraction methods, uses low temperature to reduce the oxidation of polyunsaturated fatty acids, decreases residual solvent contaminants (polychlorinated biphenyls and heavy metals), does not modify the biomass, and allows other bioactive molecules to recover. The ethanol used as a co-solvent is much more food-compatible than hexane [207]. The disadvantage of this method is the extract’s smell due to volatile compounds. Encapsulation decreases this effect and improves the extracts’ palatability [208].

##### Possible Uses of the Recovered Omega-3

Omega-3 fatty acids are employed in supplements and fortified food and feed [209]. Moreover, they can be used to ameliorate cutaneous abnormalities and maintain skin homeostasis in cosmetics [210]. 

### 4.3. Secondary Metabolites Recovered from Agri-Food

#### 4.3.1. Phenolic Compounds from Biowaste

Phenolic compounds (PC) are secondary metabolites characterized by an aromatic ring with more hydroxyl substituents. Plants produce them in response to environmental stimuli. They vary greatly by type and level based on target species and biotic and abiotic factors. Biocontrol agents, such as *Trichoderma* strains, can interfere with phenolic production in plants [11,13,65,211,212]. Phenolic compounds have antioxidant and antimicrobial properties [8] and beneficial potential for human health, such as antitumor, anti-obesity, anti-inflammatory, and ultraviolet (UV) radiation protective activities. Isolated and dosed phenolic compounds [213] were used to prepare food supplements [9,67], nutricosmetic formulations [10,64,71], and nutraceutical food [66,214]. The process used to recover PC from biowaste consists of a pre-treatment (thermal treatment of the sample followed by electro-osmotic dewatering, foam mat, pulverization, or micro-filtration) [215], extraction (obtained by maceration, or assisted by microwave, ultrasound, supercritical fluid, pressurized liquid, pulsed electric field, high voltage electrical discharges, or multi-technique) [216], concentration (obtained by distillation, or steam distillation), and purification (obtained by microfiltrations, ultrafiltration, resins, or chromatography) [216]. 

##### Anthocyanins from Agri-Food Waste

Anthocyanins are water-soluble antioxidants pigments that belong to the flavonoid family. They have a C6-C3-C6 skeleton [217]. The flavylium cation gives the red color to the anthocyanins [218]. Temperature, enzymes, pH, light, metallic ions, oxygen, sulfites, and interaction with flavonoids, phenolics, ascorbic acid, and sugars can affect anthocyanins’ integrity according to their chemical structure and food concentration. Anthocyanins subjected to high temperatures and prolonged heating oxidize. Moreover, the deglycosylation process, nucleophilic attack of water, cleavage, and polymerization determine the structure breakdown. These structural changes decrease anthocyanin content in the final product [217]. Sulfite adds colorless adducts interrupting the conjugated π-electron system [218]. Anthocyanin association reactions (e.g., self-association between anthocyanins via hydrophobic interactions, co-pigmentation between anthocyanins and other phenols through van der Waals interactions between the planar polarizable nuclei or binding the sugar and the flavylium nucleus covalently, and metal complexing between anthocyanins and with metals, via their o-hydroxy groups) defend the flavylium chromophore from the water’s nucleophilic bond, enhancing their stability and color [219]. The anthocyanins can manage and/or prevent chronic degenerative diseases, including cancers, cardiovascular diseases, type 2 diabetes mellitus, dyslipidemias, and neurodegenerative diseases [220]. Several biochemical parameters involved in the inflammatory responses (e.g., interleukins (ILs), tumor necrosis factor-alpha (TNF-α), nuclear factor-kappa B (NF-kB), and cyclooxygenase 2), which are able to improve malonaldehyde and reactive oxygen species, and to reduce the activity/expression of antioxidant enzymes (e.g., catalase and superoxide dismutase), have been related to the prevention or development of these diseases [221,222,223]. The anthocyanins improve the sensitivity, secretion, and lipid profile of insulin by inhibiting the limiting enzyme of cholesterol synthesis (3-hydroxy-3-methylglutaryl-coenzyme A) or adipose triglyceride lipase engaged in triglyceride breakdown in diabetics and prediabetic subjects [224,225]. Moreover, anthocyanins regulate the expression of adipokines, thereby enabling the avoidance of insulin resistance and the progression of type 2 diabetes mellitus [226]. Anthocyanins can reduce triglycerides, cholesterol, LDL-cholesterol, and inflammatory biomarkers regulating the expression/activity of pro-inflammatory cytokines (e.g., IL-6, TNF-α, and IL-1A), pro-inflammatory enzymes (e.g., COX-2), and NF-κB signaling pathways, decreasing the production of pro-inflammatory molecules (e.g., C-reactive protein), and improving SOD and proliferator-activated receptor-γ expression [227,228]. Finally, anthocyanin supplementation can enhance cardiovascular function [229,230], regulate gut microbiota composition [231,232], improve exercise recovery effectiveness [233], decrease ulcerative colitis symptoms [234], reduce ocular fatigue [235], and promote healthy facial skin conditions [233]. Some innovative processes are proposed for the extraction of anthocyanin from biowastes, such as the extractions assisted by a pulsed electric field, microwave, and ultrasound technologies. The enhancement of the extraction of anthocyanin, helped by pulsed electric field technology, is related to permanent (irreversible) or temporary (reversible) pores in the cell membranes, which facilitate the anthocyanins release into the medium [217]. The target compounds’ extraction does not constantly improve with the increasing of the electric field strength, specific energy input, treatment time, temperature, and pulse number [236,237]. For example, studies on blueberry extraction showed an improvement (+75%) in the extraction of anthocyanin when the specific energy input was increased [238]. Instead, studies showed that improving electric field intensity and specific energy input does not increase the anthocyanins content extracted from the sweet cherry byproduct [239]. The application of high intensity can lead to anthocyanin degradation. instead, the application of low/moderate-intensity enhanced anthocyanin recovery without anthocyanin’s degradation/modification. The pulsed electric field technology allows the selective extraction of the single anthocyanin classes [240]. The pulsed electric field technology improves the monoglucoside anthocyanin extraction compared to acylated glucoside anthocyanins from grape pomace [241] and the extraction of cyanidin, delphinidin, and petunidin glycosides from blueberry byproducts [242]. The anthocyanin recovery from grape pomace enhances when the microwave and irradiation time improve [243], and longer irradiation times enhance anthocyanin recovery from wine lees [243], sour cherry pomace [244], and saffron floral bio-residues [245]. Nevertheless, the excessive intensification of MW extraction process parameters can decrease the extraction of anthocyanin from biowaste due to their degradation (anthocyanins are thermolabile compounds) [149]. Thus, it is recommended to use extraction temperatures below 60 °C to minimize the anthocyanin’s losses. The extraction time also impacts MW extraction. The excess time causes the degradation of anthocyanin due to higher exposure to microwave powers and high temperatures [246]. The high microwave power determines internal overheating, leading to carbonization and isomerization, and/or degradation of molecules [129]. According to some authors, anthocyanins’ thermal degradation determines the loss of sugar moieties, the formation of a carbinol pseudo base, and chalcone by hydrolysis of the remaining sugar moiety and cut between C2 and C3 [237]. According to others, the degradation of anthocyanin is due to decomposition reactions of water molecules and the production of reactive oxygen species [247]. Studies on anthocyanin recovery from eggplant peel and fig peel showed that the recovery of anthocyanin decreased when the microwave powers and irradiation times improved [248]. Studies on grape pomace [240], blackcurrant bagasse [249], blueberry peel [250], black rice bran [251], and corn husk [252] showed that the extraction of anthocyanin decreased when the irradiation times were long. Finally, anthocyanins’ structures impact their recovery from bio matrices. For example, anthocyanin analogs that are unsubstituted at C3 of the C-ring are more stable to MW treatment than other anthocyanins [253], as well as acylated anthocyanins than non-acylated ones [254]. Another technique used to improve the extraction of anthocyanin is ultrasound. Acoustic cavitation can determine the thermal and chemical degradations of the anthocyanins since the acoustic cavitation phenomenon can determine thermal stress and free radical formation [255]. Long processing times can cause severe degradations in anthocyanins [256]. For example, the anthocyanin extracted from black chokeberry wastes degrades when an ultrasound water bath (30.8 kHz) for 60 min at 70 °C, with a nominal ultrasound power of 100 W, and 50% ethanol in water are used [257]. Using enzymes (pectinase compound and pectinase) combined with ultrasound can improve the extraction technique’s performance [256]. 

##### Possible Uses of the Recovered Phenolics

Phenolics are used as functional food additives. Their antimicrobial and antioxidant activities enhance the shelf-life of foodstuffs [258]. Anthocyanins can be employed as a coloring additive (EFSA code E163).

Phenolic compounds can be employed as supplement ingredients [259], pharmaceutical [258], and cosmeceutical agents [71]. 

Caffeic and gallic acids can be used in chitosan-based biofilms to inhibit the growth of *Bacillus subtilis* and *Staphylococcus aureus* and enhance the film’s oxygen and vapor permeability [260].

Tannins could develop protein-based biofilms since they can interact with proteins through non-covalent bonds and hydrogen bonding [261]. 

Finally, the phenolics might be helpful to the textile industry as natural dyes with antimicrobial properties [261]. 

#### 4.3.2. Carotenoids from Agri-Food Waste

Carotenoids are fat-soluble pigments with excellent antioxidant activity (e.g., singlet oxygen-quenching capacity and free radical activity) [262,263]. The two main classes of carotenoids are xanthophylls (yellow color) that contain oxygen and carotenes (orange color) that consist of linear hydrocarbons, which can cyclize at both ends of the molecule [71]. Carotene supplementation is related to the inhibition of atherosclerosis-related multiple sclerosis [264], cardiovascular diseases [265], macular degeneration [266], and degenerative diseases [267]. Carotenes are most bioavailable in their natural trans-form [268,269]. Light, metals, heat, and pro-oxidants can isomerize the trans-form into cis-form [270]. The extraction of carotenoids is achieved using organic solvents (e.g., hexane, methanol, acetone, and ethanol or solvent combinations). The supercritical fluid extraction with CO_2_ improves the extraction’s efficiency, as is also the case for the extraction of carotenoids from carrot peels (+86.1% at 349 bar) [271]. In addition, the microwave [272] and ultrasound increase the carotenoids’ recovery [273]. Finally, an extraction method of lycopene and pectin from tomato pomace [274] and pink guava decanter [275], which relied on a simple water-induced hydrocolloidal complexation, was tested. The pH, temperature, solid loading, and stirring affected the complexation of carotenoids and pectin [276]. 

##### Possible Use of the Recovered Carotenoids

Carotenoids can be used as food and feed additives. Supplementation of the animal diet with carotenoids improves the nutritional quality of animal products [277]. 

Moreover, they can be employed as a food colorant to improve food desirability and acceptability [278]. 

#### 4.3.3. Essential Oil from Agri-Food Waste

Essential oils are lipophilic substances, including terpenic hydrocarbons (e.g., monoterpenes and sesquiterpenes) and oxygenated derivatives (e.g., aldehydes, phenols, esters, and alcohols) [279]. The traditional methods used to extract the essential oils from plant matrices are liquid–solvent extraction and steam- and hydro-distillation. High temperatures (around 100 °C) can lead to the decomposition of essential oils, and the use of pentane and hexane can determine toxic organic residues. Supercritical fluid extraction (using carbon dioxide) is the most widely applied innovative technological process. Carbon dioxide is suitable for the extraction of lipophilic compounds since it has a polarity similar to pentane as well as other desirable characteristics such as being non-flammable, non-toxic, available in high purity, and easily removed from the extract, as well as having low temperature (32 °C) and critical pressure (74 bar). Favorable extraction conditions are related to the solubility of essential oil compounds in supercritical CO_2_ [264], the plant’s pretreatment (used to facilitate the extraction by improving solvent contact and breaking cells), and online fractionation (used to achieve the separation of the essential oil from cuticular waxes) [280]. Essential oils from *Lamiaceae* (e.g., oregano, thyme, rosemary, sage, basil, and marjoram) and citrus (e.g., lemon, orange, etc.) family plants [280] were extracted using this technology. The fractionization, which can be achieved in the mode of multi-step fractionation (successive steps in which CO_2_ density improves) or online fractionation (using a cascade decompression system consisting of two or three separators in series), can improve the selectivity of supercritical fluid extraction. This strategy can be employed when molecules with different solubilities in supercritical CO_2_ are extracted from the same matrix [280]. 

##### Possible Uses of the Recovered Essential Oils

Essential oils can be used as flavors, fragrances [281], and antimicrobial agents [282] in food and cosmetic products. 

Moreover, they can be incorporated into food packaging to improve the UV barrier property and surface hydrophobicity to protect foods against oxidation and microbial injuries [283]. 

Finally, they can be used as green pesticides in agriculture [284]. 

#### 4.3.4. Organosulfur Compounds from Agri-Food Waste

Organosulfur compounds are thiols with sulfur in their structure. They include glucosinolates (isothiocyanates), allyl sulfides, indoles, and sulforaphane [285]. Glucosinolates are thioglucosides that are produced in cruciferous vegetables of the Brassica family (e.g., broccoli, radish, cabbage, and cauliflower). They are responsible for the plant’s defense against insects and pathogens. Moreover, they have some health-beneficial properties, including antioxidant (e.g., scavenge free radicals) [286], anti-inflammatory (e.g., activating detoxification enzymes, suppression of interferon regulatory factor 3, and macrophage migration inhibitory factor) [287,288], cardioprotective (e.g., they reduce low-density lipoproteins), neuroprotective, and anti-carcinogenic attributes (detoxifying carcinogens and toxicants) [289,290]. The conventional technologies used to extract them employ boiling water or aqueous organic solvent extraction [291] in a single extraction process or repetitive cycles [292]. Among the non-conventional extraction technologies, ultrasound techniques (20 kHz and 400 W) were used to improve the extraction’s yield of the sinigrin from Indian mustard (+70.67% recovery than conventional techniques), microwave techniques were sued to decrease the extraction time of sulforaphane from cabbages (30 min for conventional extraction to 1.5–3 min for microwave-assisted technique) [293,294], and supercritical technology was used to extract allyl isothiocyanates from wasabi (SC-CO_2_, 35 °C, and 25 Mpa) [295]. 

Allyl sulfides, produced by alliaceous vegetables (e.g., shallots, chives, leeks, and scallions), originate from S-alk(en)yl-L-cysteine sulfoxides and γ-glutamyl-S-alk(en)yl-L-cysteines. Among these, diallyl sulfides are responsible for the pungent aroma [296,297]. Organosulfur compounds have some health-beneficial properties, including anticancer (e.g., they promote apoptosis, xenobiotic-metabolizing enzyme production, and carcinogen detoxification, in addition to the production of the enzymes that are responsible for DNA repair, are engaged in cell cycle arrest, and decrease the metabolism of nitrosamines and hydrocarbons), antioxidant (e.g., they scavenge free radicals), and antimicrobial properties [298,299]. They are thermally unstable and are lost during sterilization, pasteurization, drying, and cooking [300]. High-temperature processing decreases their bioavailability [301,302], while high-pressure processing reduces their anticancer, antimicrobial, and antioxidative properties, decreases their enzyme alliinase activity [303] and improves their enzyme alliinase activity [304,305]. Moreover, freeze-drying and infrared-drying technologies [306] and microwave-assisted and pressurized liquid extractions have negative thermal effects. Therefore, only supercritical fluid extraction is suitable for efficient extraction among the new technologies used to replace conventional ones [307]. 

##### Possible Uses of the Recovered Organosulfur Compounds

Organosulfur compounds can be used as supplements, food additives (because of their aromatic taste and smell) [308], and biopesticides [309]. 

## 5. The Circular Economy and Biowaste Recycling Strategies

**Figure 1:**
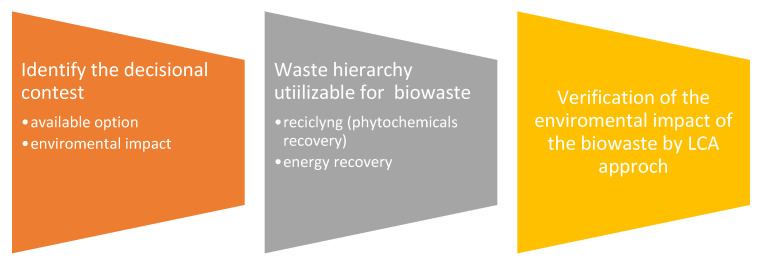
Methodological approach to finding the environmentally preferable option for bio-waste management.

Several studies were performed to evaluate the potential of biowaste and byproducts for circular use. Coherently with the European Waste Framework Directive [1], the primary and critical principle for identifying the hierarchy of actions is reducing the loss of high-value resources and promoting the growth of bio-materials, which are reusable for other purposes. Some elements may limit the applicability of the valorization route, such as environmental impact, technical innovation, economical productivity, and legislation compliance (Figure 1).

**Figure 2:**
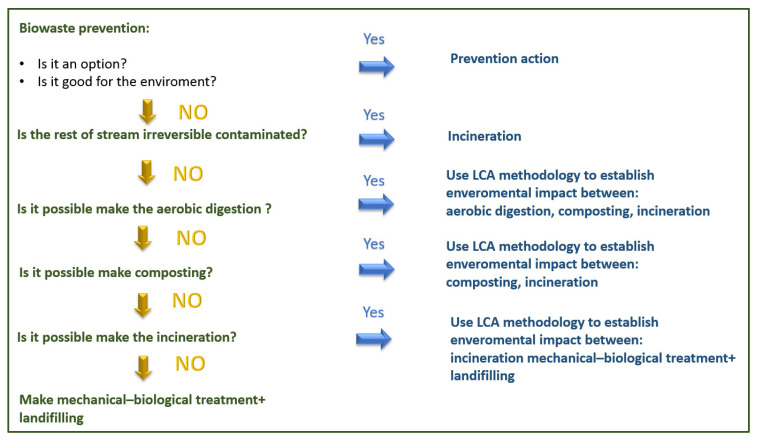
List of guidelines to make valid environmental decisions for the management of organic waste.

Robust assessment tools are necessary to make the right choices during the design of recycling processes in order to reduce their environmental impacts [310] (Figure 2). 

The extraction of bioactive compounds (phytochemicals) is workable when a homogeneous material stream is available (e.g., fruit and vegetable are considered a rich source of antioxidants, phenols, carotenoids, polyphenols, and fibers) [311].

In contrast, the phytochemicals are scarcely obtainable in the case of heterogeneous streams, as is the case in food waste produced at the consumption stage [310]. Technologies that imply less dissipative use of the bio-based resources must be preferred. For example, anaerobic digestion is considered better than composting to produce methane [312]. Moreover, not all the solutions proposed by the scientific community to allow the circularity of organic waste are applicable on an industrial scale.

Measuring the environmental impact of different economic value chains is essential for the simplification of evidence-based policymaking. The Life Cycle Assessment (LCA) methodology is considered a suitable approach for comparing different biowaste management options. LCA considers all processes from the extraction of the bioactive compounds to the end-of-life [313]. One of the most important benefits of this approach is that it considers the burden-shifting (transfer of environmental influences between supply chain phases or environmental sections) that may occur when pushing for resource efficiency. It is worth noting that the hierarchy could be modified according to the sustainability priorities.

Nevertheless, the impact assessment and product system modeling limit the quantitative estimation of the environmental aspects. Only a few data points are known regarding innovative recovery processes, and thus, the setting of the system limits may not be facile, and the allocation of impacts may be variable [313]. Therefore, the robustness and comprehensiveness of LCA must be improved before applying it to bio-based and circular systems.

## 6. Conclusions

A world without waste is the main objective of the Circular Economy Package proposed by the European Parliament and Council in 2018. Some proposals have been made to manage biowaste, including making biochar and bioenergy (e.g., biogas, biodiesel) or recovering bioactive molecules for cosmetic, pharmaceutical, and food supplements. The low disposal cost compared to the recycling/conversion cost proved to be the main problem to be addressed. This work highlights the importance of looking for new technologies with competitive costs, low environmental impact, and usability in the industrial process. Even if much has been achieved, significantly more still needs to be studied to achieve the goal.
